# On the Respiratory Mechanics Measured by Forced Oscillation Technique in Patients with Systemic Sclerosis

**DOI:** 10.1371/journal.pone.0061657

**Published:** 2013-04-26

**Authors:** Ingrid Almeida Miranda, Alvaro Camilo Dias Faria, Agnaldo José Lopes, José Manoel Jansen, Pedro Lopes de Melo

**Affiliations:** 1 Biomedical Instrumentation Laboratory, Institute of Biology and Faculty of Engineering, State University of Rio de Janeiro, Rio de Janeiro, Brazil; 2 Laboratory of Pulmonary Function Testing – Discipline of Pneumology, Faculty of Medical Sciences, State University of Rio de Janeiro, Rio de Janeiro, Brazil; 3 Laboratory of Clinical and Experimental Research in Vascular Biology - Biomedical Center, State University of Rio de Janeiro, Rio de Janeiro, Brazil; Harvard School of Public Health, United States of America

## Abstract

**Background:**

Pulmonary complications are the most common cause of death and morbidity in systemic sclerosis (SSc). The forced oscillation technique (FOT) offers a simple and detailed approach to investigate the mechanical properties of the respiratory system. We hypothesized that SSc may introduce changes in the resistive and reactive properties of the respiratory system, and that FOT may help the diagnosis of these abnormalities.

**Methodology/Principal Findings:**

We tested these hypotheses in controls (n = 30) and patients with abnormalities classified using spirometry (n = 52) and pulmonary volumes (n = 29). Resistive data were interpreted with the zero-intercept resistance (Ri) and the slope of the resistance (S) as a function of frequency. Reactance changes were evaluated by the mean reactance between 4 and 32 Hz (Xm) and the dynamic compliance (Crs,dyn). The mechanical load was evaluated using the absolute value of the impedance in 4 Hz (Z4Hz). A compartmental model was used to obtain central (R) and peripheral (Rp) resistances, and alveolar compliance (C). The clinical usefulness was evaluated by investigating the area under the receiver operating characteristic curve (AUC). The presence of expiratory flow limitation (EFL) was also evaluated. For the groups classified using spirometry, SSc resulted in increased values in Ri, R, Rp and Z4Hz (p<0.003) and reductions in Crs,dyn, C and Xm (p<0.004). Z4Hz, C and Crs,dyn exhibited a high diagnostic accuracy (AUC>0.90). In groups classified by pulmonary volume, SSc resulted in reductions in S, Xm, C and Crs,dyn (p<0.01). Xm, C and Crs,dyn exhibited adequate diagnostic accuracy (AUC>0.80). It was also observed that EFL is not common in patients with SSc.

**Conclusions/Significance:**

This study provides evidence that the respiratory resistance and reactance are changed in SSc. This analysis provides a useful description that is of particular significance for understanding respiratory pathophysiology and to ease the diagnosis of respiratory abnormalities in these patients.

## Introduction

Systemic sclerosis (SSc), also known as scleroderma, is a rare, chronic and multisystemic connective tissue disease characterized by fibrosis and vascular abnormalities in the skin, joints and visceral organs [1], [2], [3]. SSc affects approximately 75,000–100,000 individuals in the United States [1], and it is the most severe among the connective tissue diseases [4]. The main organs affected by SSc are the kidneys, heart and lungs [5].

The main pulmonary manifestations that appear during the course of SSc include pulmonary arterial hypertension and/or lung fibrosis [2], [6]. The incidence of lung diseases varies according to the population and diagnostic methods used, and it can reach up to 90% for interstitial lung disease and 50% for pulmonary hypertension, as observed in autopsy studies [6], [7], [8]. In patients with SSc, the presence of any of these lung diseases is associated with reduced survival [3], [9]–[13], and the main cause of death for the patients with SSc is the associated lung disease [2], [5], [13]–[16]. Lung affection is also a determinant of the quality of life and morbidity of these patients [7], [8]. The largest study in the literature included more than 1,000 patients, and lung affection was the cause of 33% of the deaths [7], [17]. The reduction of the lung volume is the most common ventilatory disorder in these patients [8], [11], [12], [18].

Several studies have already shown that pulmonary function tests are recommended routinely in the follow-up of all the patients with SSc, especially during the first four years of the disease [8], [9], [11], [13]. Therefore, modern techniques to assess the pulmonary function and imaging exams were used to evaluate the lung structure in the earlier stages of SSc [19].

### Resistive and reactive properties of the respiratory system

The forced oscillation technique (FOT) is a non-invasive method that allows for the assessment of the resistive and reactive properties of the respiratory system [20], [21], [22]. This method consists of an application of sinusoidal signals during normal respiration by means of an external pressure generator, allowing the measurement of the respiratory system impedance (Zrs). The main advantages of FOT are simplicity in the performance of tests, which require little cooperation by the patients, production of parameters complementary to the traditional methods for pulmonary assessment and reduced time to perform the tests [20], [21], [22]. Although it has been successfully used in several diseases [20]–[26], there are no studies assessing the potential of this technique for the evaluation of pulmonary function in patients with systemic sclerosis.

### Expiratory flow limitation

The analysis of inspiratory and expiratory impedance separately provides useful information to detect expiratory flow limitation (EFL) [27]–[29]. Under normal conditions, low-frequency reactance measurements reflect the elastic properties of the entire respiratory system. However, when EFL is present, the oscillatory signal cannot pass through the choke point and reach the alveoli, and the reactance reflects the mechanical properties of airways proximal to the choke point [27], which are much stiffer than those of the periphery. This results in a marked reduction of respiratory reactance, when measured using low-frequency FOT. As a result, FOT can reliably detect EFL by intra-breath variations in Xrs [22], [27]. Recently, Dellacà et al. were the first to use this method to obtain new data concerning the EFL and response to salbutamol in patients with COPD [27]–[29]. Previous studies from our group showed promising results using inspiratory and expiratory (within-breath) analysis to investigate the pathophysiology of asthma [30] and COPD [31].

In patients with restrictive diseases, the maximal expiratory flows are usually well preserved but there is a marked decrease in functional residual capacity. As a result, these patients breathe at low lung volumes (ie, near residual volume), where the maximal expiratory flows are relatively small. It was recently postulated that under these conditions, there could be EFL in these patients [32], [33]. The presence of EFL during resting breathing in patients with SSc has not been explored.

Therefore, the purpose of the present study was threefold, as follows: (1) to investigate the influence of SSc on the resistive and reactive properties of the respiratory system, (2) to evaluate the clinical potential of the FOT indices in the diagnosis of respiratory alterations due to SSc and (3) to analyze the EFL during resting breathing in these patients. These analyses were performed using as a reference the two most common methods currently used for the diagnosis of respiratory disorders in SSc, namely, spirometry and lung volume assessment.

First, in a multi-frequency study, we investigated the influence of restrictive characteristics, as determined by reductions in spirometric and lung volumes parameters, on the respiratory resistance and reactance of SSc patients. A compartmental model is also included in this analysis. Next, the associations between FOT and spirometric parameters and lung volumes were evaluated. Then, the ability of the FOT to detect respiratory alterations in SSc patients was analyzed. Finally, in a mono-frequency study, we analyze the EFL in these patients.

## Materials and Methods

### Study design

This report describes a controlled observational study assessing prevalent cases, where individuals were the unit of assessment. Tests included measurements of FOT, spirometry and lung volumes. The measurements were performed at the Pulmonary Function Testing Laboratory of the Pedro Ernesto University Hospital (HUPE) and at the Biomedical Instrumentation Laboratory of the State University of Rio de Janeiro.

The study protocol complied with the guidelines of the Declaration of Helsinki and was approved by the HUPE ethical committee. Before performing the tests, all the participants were duly informed as to the content of the tests and signed an informed consent form.

### Selection of individuals

We used the two most widely used methods to assess the pulmonary function of SSc patients as a reference to classify these individuals, spirometry and lung volumes.

### Analysis using spirometry as reference

A total of 82 individuals were selected, from which 52 had systemic sclerosis, and 30 were healthy and represented the control group. The individuals with systemic sclerosis were divided into the following two groups: the normal to the exam group (n = 22) consisted of individuals diagnosed with SSc and normal spirometry, and the restrictive group (n = 30) was composed of individuals diagnosed with SSc and associated restrictive ventilatory disorder by spirometry [34], [35].

### Analysis using lung volume exams as reference

The lung volumes were measured by means of the helium dilution technique [36]. A total of 50 volunteers were assessed, including 29 individuals with SSc and 21 healthy individuals. The patients with SSc were divided into the following two groups: one comprised patients whose results were normal (n = 7), and the other comprised individuals diagnosed with pulmonary restriction (n = 22).

The patients with SSc were assisted at the rheumatology outpatient clinic of HUPE, and they were diagnosed following the criteria of the American College of Rheumatology [37]. The control group comprised HUPE staff and students at UERJ.

### Eligibility criteria

The criteria for inclusion in the present study were a confirmed diagnosis of systemic sclerosis according to the criteria of the American College of Rheumatology [37] and individuals from both genders. The exclusion criteria were a history of smoking, exacerbation of disease in the previous 90 days, presence of chronic lung diseases, tuberculosis or pneumonia, presence of chest trauma or surgery, respiratory infections in the previous 30 days, chemotherapy and/or radiotherapy for cancer and an inability to perform the tests.

The control group included healthy volunteers from both genders without a history of lung or cardiovascular disease or smoking. These individuals exhibited normal spirometry [38], [39] and an absence of respiratory infections.

### Protocol for the exams

The patients stopped the use of bronchodilators 12 hours before performing the tests and did not drink coffee and/or alcohol during the previous six hours. All patients were evaluated between 8 and 10 in the morning. The order in which the tests were performed was the following: initial anamnesis to obtain anthropometric measurements, FOT exams, spirometry and helium dilution.

### Instrumentation

To assess the respiratory mechanics, pressure oscillations were applied within the frequency range of (4–32 Hz) with an amplitude of approximately 1 cmH_2_O that was produced by a loudspeaker coupled to the respiratory system by means of a mouthpiece. The resulting flow and pressure signals were measured near the mouth by means of a pneumotachograph and a pressure transducer, respectively. From these signals, 4 primary data blocks of 4 s and 3 secondary blocks obtained by 50% overlapping between primary blocks were multiplied by a Hanning window and then processed by Fourier analysis. This way, the respiratory system impedance (Z_rs_(f)) was estimated by equation (1). 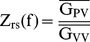
(1)


Where 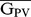 and 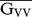 are the mean crosspectrum between pressure and flow and mean autospectrum of flow, respectively. The impedance values resulted from the average of three determinations.

The interpretation of the multifrequency respiratory impedance data and its association with the mechanical and structural properties of the respiratory system was performed using classical FOT parameters [20]–[22], [40]–[50]. Analysis of a linear regression in the resistive component of the impedance in the frequency range between 4 and 16 Hz was used in order to achieve intercept resistance (Ri) and the slope of the resistive component of the impedance (S). Resistances measured between 4–32 Hz are related to the airway and tissue Newtonian resistance plus the delayed airway resistance resulting from gas redistribution. Ri is an extrapolation to the intercept, estimating how the cited properties work at low frequencies. Thus, this parameter does not include tissue viscoelastic properties that are manifested below 4 Hz. S reflects the frequency-dependent alteration in the distribution of gas flow within the system, i.e. both spatial and temporal inhomogeneity [41]–[43], [51]. In healthy adults, S presents values near zero, while more negative are observed in patients [20]–[22], [40]–[50]. The mean resistance (Rm) was also calculated for the frequency range between 4 and 16 Hz. This parameter is related to the airway caliber [40].

The imaginary component of Zrs (Xrs) was used to evaluate the parameters related to accumulation of energy in the respiratory system. The analysis in a frequency range of 4–32 Hz was used to measure the resonant frequency (fr). The inertive component of Xrs demonstrates a positive phase shift, i.e. the pressure leads the flow. However, the compliant component of Xrs shows a negative phase shift, i.e. the pressure lags the flow. When the magnitudes of these shifts are equal, then Xrs becomes zero, and resonance occurs [41]–[43], [51]. The frequency at which resonance occurs is termed the resonant frequency (fr). This parameter reflects changes in airway heterogeneity, which reduces dynamic compliance, as well as tissue changes associated, for example, with the presence of fibrosis. Two other parameters were used to obtain a detailed characterization of the properties of energy accumulation, the respiratory system mean reactance (Xm) and dynamic compliance (Crs,dyn). Xrs was measured in the 4–32 Hz range and is usually related to respiratory system non-homogeneity [44]. Crs,dyn evaluated by the FOT is measured from the mouth, including the effects of the lung and airway wall compliances, the compliance of the chest wall/abdomen compartment and thoracic gas compression. This parameters was estimated in relation to the respiratory reactance at an oscillatory frequency of 4 Hz (X4Hz) and using the equation X4Hz = −1/(2πfCrs,dyn) [45]. The same frequency was used to evaluate the absolute value of the respiratory impedance (Z4Hz), which is obtained using respiratory system resistance (R4Hz) and reactance (X4Hz) as described in equation (2):
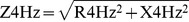
(2)


This parameter is associated with the work performed by the respiratory muscles to overcome resistive and elastic loads, promoting the movement of air in the respiratory system [40]. It is related with fatigue and breathlessness, one of the most important symptoms in predicting quality of life in respiratory patients.

Three multi-frequency FOT exams were performed, which lasted approximately 16 seconds and were separated by one-minute intervals. The exams were rated technically appropriate when a minimum of 0.9 in the coherence function was achieved [23], [24], [41]. The final result of the tests was estimated by calculating the average of three satisfactory exams. Before performing the tests, the individuals remained coupled to the device under spontaneous ventilation for approximately one minute to adapt to the device and the testing environment. While performing the exams, the individuals remained seated with the chest straight and the head in a neutral position relative to the device, and they wore a nose clip to prevent air escape. The individuals breathed calmly through a silicone mouthpiece while holding the cheeks and the lower part of the chin with the hands to minimize the shunt effect [20], [21], [22].

The devices used in spirometry were Vitatrace VT 130 SI (Pró-médico, Rio de Janeiro, Brazil), Collins/GS (Warren E. Collins, Inc., Braintree, Massachusetts, United States) and NSpire Health (NSpire Health, Inc., Louisville, Kentucky, United States). The lung volume tests were performed using the Collins/GS device. The tests followed the Guidelines for Pulmonary Function Tests [27]. The following spirometric parameters were assessed: forced vital capacity (FVC); forced expiratory volume in one second (FEV_1_); FEV_1_/FVC ratio; forced expiratory flow at 25–75% of FVC (FEF_25–75%_); and the FEF/FVC ratio.

To assess the lung volumes by means of the helium dilution technique, the total lung capacity (TLC), residual volume (RV) and the RV/TLC ratio were analyzed. All the values were assessed in absolute and percent terms relative to the expected values for gender, age and height according to the protocol by Pereira *et al.* [52] and Knudson *et al.* [38] and complying with the criteria established by the American Thoracic Society [53].

### Compartmental model analysis

**Figure 1:**
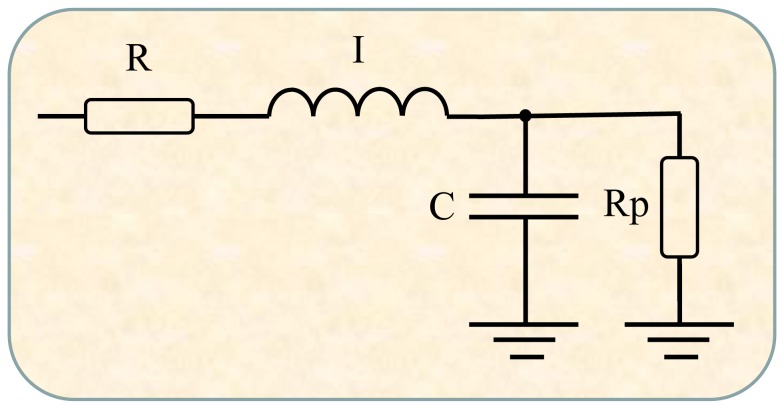
Electrical representation of the two-compartment model used to analyze respiratory impedance. Resistance (R), inductance (I) and capacitance (C) are the analogs of mechanical resistance, inertance and compliance, respectively. This model contributes to gain additional insight into anatomical or pathophysiological changes in the studied subjects allowing the evaluation of central (R) and peripheral (Rp) airway resistance.

To gain additional insight into anatomical or pathophysiological changes in the studied subjects, we applied a compartmental model to the multi-frequency impedance data. To this end, we used the extended RIC (eRIC) model (Figure 1) where R is analogous to central airway resistance and Rp describes peripheral resistance, I is associated with lung inertance and C with alveolar compliance [54]. This model is proposed as an improvement to the basic RIC model [21]. Specifically, the added peripheral resistance Rp allows for the frequency dependence observed of typical real impedance data, which is beyond the RIC model's capability. The physiological justification for this additional component is that it describes the resistance presented by the respiratory system's small airways.

Model parameters were estimated using the Levenberg-Marquardt algorithm to determine the set of coefficients of the nonlinear model that best represents the input data set in the least- squares sense. Along with the corresponding model estimates, this analysis also provides the evaluation of the total error value, an overall measure of “goodness of fit” for the model. This parameter is defined herein as the square root of the sum of the real and imaginary impedance estimation errors.

### Expiratory Flow Limitation Analysis

This study was performed in eighteen stable SSc patients. The patients met the standard diagnostic criteria for SSc [37] and were current or light smokers. It was a mono-frequency study in which Xrs was measured at an oscillation frequency of 6 Hz. The peak-to-peak oscillation was 2.0 cmH_2_O. The signals were sampled at 1024 Hz and low-pass filtered at 25 Hz to remove noise. During post-acquisition data processing, the breathing signal was extracted using a 4^th^ order 2 Hz low-pass filter and the 6 Hz oscillation signal was extracted using a 6^th^ order ±1 Hz band pass filter centered around 6 Hz. Any exam with a coherence value *<*0.90 was excluded. Two FOT recording were performed over 30 s during stable breathing, while subjects used their hands to support their cheeks and wore a nose clip. Fourier coefficients at 6 Hz of pressure and flow were computed and combined according to equation (1) to obtain Xrs. This analysis used thirty primary data blocks of 1 s and 29 secondary blocks obtained by 50% overlapping between primary blocks, providing a time resolution of 0.5 s.

Breathing cycles containing artifacts due to leaks and glottic closures, as well as incomplete or partial breaths, were removed from the analysis. The total number of breaths remaining after analysis was calculated. The expiratory flow limitation index (EFLi) was calculated as the difference between the mean inspiratory Xrs and expiratory Xrs values of each breath. According to [27], [55], we considered that Expiratory flow limitation was present when the EFLi index was *>*2.8 cmH_2_O/L/s.

### Presentation of the results and statistical analysis

Statistical analyses were performed using the software Microcal^(^™^)^ Origin® 6.0 (Microcal Software Inc., Northampton, Massachusetts, United States), STATISTICA® 5.0 for Windows (StatSoft Inc., Tulsa, Oklahoma, United States), and MedCalc® 10.0 (MedCalc Software, Mariakerke, Belgium). First, the characteristics of the sample distribution were assessed by means of the Shapiro-Wilk test. When samples exhibited a normal distribution (parametric behavior), the one-way ANOVA was applied for intergroup analysis followed by Tukey's test to compare among several groups. When the distribution exhibited a non-normal character (non-parametric), the Kruskal-Wallis ANOVA was applied for intergroup analysis, and the Mann-Whitney test was used to compare among several groups. To analyze the agreement between the spirometric and lung volume tests, the non-parametric McNemar test was applied. Differences were considered statistically significant when p<0.05.

The Pearson's correlation coefficient was used to analyze the associations among the FOT parameters, spirometry and lung volumes, which were classified as follows: [56]

- Small or no correlation: correlation between 0 and 0.25 (or −0.25);
- Reasonable correlation: correlation between 0.25 and 0.50 (or −0.25 to −0.50);
- Moderate to good correlation: correlation between 0.50 and 0.75 (or −0.50 to −0.75);
- Very good to excellent correlation: correlation greater than 0.75 (or −0.75).

The evaluation of the diagnostic use of the FOT parameters was performed by comparing normal individuals and patients with restrictive characteristics using the software *MedCalc®* 10.0 (MedCalc Software, Mariakerke, Belgium) by means of analysis of the receiver operating characteristic (ROC) curves. The optimal cut-off point was chosen to balance the highest values of sensitivity and specificity. The parameter performance was described by the area under the ROC curve (AUC) [57].

## Results

### Investigated population

The anthropometric characteristics of the investigated groups are described in Table 1. Among all of the studied characteristics, only age exhibited a statistically significant difference between the control group and the normal to the exam and restrictive groups when using the lung volume tests as reference. None of the remaining parameters exhibited a significant difference.

**Table 1:** Anthropometric characteristics of the studied subjects in the analysis using spirometry and pulmonary volumes as a reference.

		Control (1)	Normal to the Exam (2)	Restrictive (3)	
Mail/Female	Spirometry Volumes	1/29	1/21	1/29	–
		5/16	1/6	1/21	–
Age (years)	Spirometry	49.66±13.49	49.59±14.49	46.13±12.79	ns
	Volumes	32.09±11.73	49.29±10.92	44.41±13.65	1–2,3–1
Body mass (kg)	Spirometry	59.17±8.67	60.78±11.90	59.90±13.08	ns
	Volumes	62.57±12.39	61.61±10.47	62.85±11.16	ns
Height (m)	Spirometry	1.59±5.25	1.56±3.36	1.57±6.30	ns
	Volumes	1.62±5.09	1.59±5.83	1.59±5.84	ns
BMI (kg/m^2^)	Spirometry	23.49±2.87	24.82±3.93	24.06±4.29	ns
	Volumes	23.92±4.74	24.08±3.36	24.62±3.68	ns

### Classification using spirometry

Table 2 shows the obtained results according to the classification of restriction based on the spirometric parameters. All of the parameters exhibited a significant reduction upon comparison among the investigated groups, except for the FEF/FVC ratio, which exhibited a progressive increase (Table 2).

**Table 2:** Spirometric parameters obtained in the groups studied using this exam as a reference.

	Control (1)	Normal to the Exam (2)	Restrictive (3)	
FVC (L)	3.35±0.68	2.82±0.70	1.96±0.54	1–2–3–1
FVC (%)	111.94±18.48	96.68±12.46	64.26±11.56	1–2–3–1
FEV_1_ (L)	2.79±0.56	2.35±0.58	1.69±0.45	1–2–3–1
FEV_1_ (%)	112.48±18.21	97.08±11.55	66.37±12.10	1–2–3–1
FEV_1_/FVC	92.45±10.37	83.42±4.04	86.58±5.45	1–2.3–1
FEF_25–75%_ (L)	3.25±0.97	3.02±0.91	2.52±0.89	1.2–3–1
FEF_25–75%_ (%)	117.38±37.37	112.93±27.16	88.57±28.35	1.2–3–1
FEF/FVC	98.58±28.12	110.03±23.41	134.41±47.76	1.2.3–1

### Classification using lung volumes

The results obtained according to the classification of restriction using the lung volume assessment as reference are described in Table 3. A significant reduction of volume and increase of the RV/TLC ratio were observed in the restrictive group.

**Table 3:** Pulmonary volumes obtained in the groups classified using these parameters as a reference.

	Control (1)	Normal to the Exam (2)	Restrictive (3)	
TLC (L)	4.42±0.86	4.64±1.01	3.09±0.69	1.2–3–1
TLC (%)	89.71±19.39	98.0±11.06	64.68±9.68	1.2–3–1
RV (L)	1.35±0.46	1.77±0.42	1.05±0.27	1–2–3–1
RV (%)	90.43±34.74	108.0±18.81	65.86±15.80	1.2–3–1
RV/TLC	31.19±9.75	38.14±4.02	34.18±6.87	1–2.3.1

### Forced oscillations

**Figure 2:**
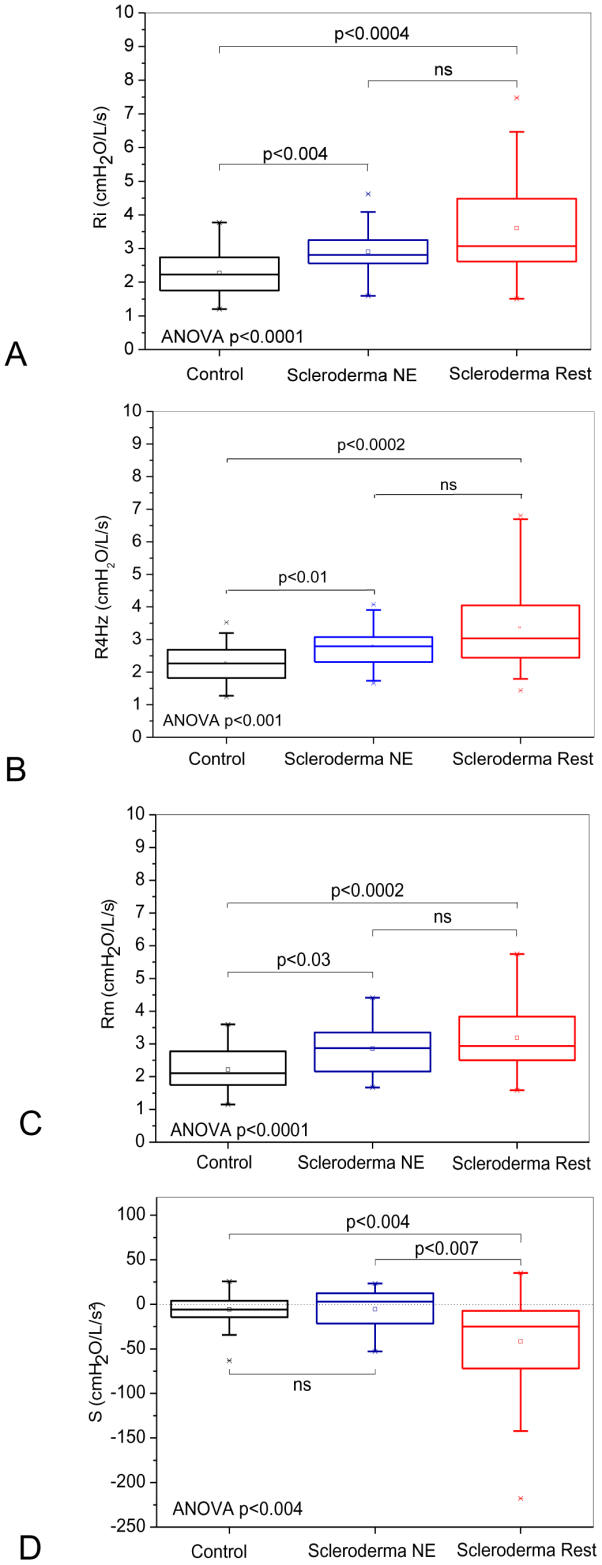
Comparison of the resistive parameters obtained from groups of patients classified according to the spirometric exams. Analyses using the forced oscillations technique showed that the total resistance of the respiratory system (Ri; Figure A) and the mean resistance (Rm; Figure B) increased in the individuals with restrictive disorder based on the spirometric test and in the patients classified as normal. The restriction resulted in more negative S values, which denote the presence of non-homogeneities in the respiratory system of these patients (Figure C). The top and the bottom of the box plot represent the 25^th^- to 75^th^-percentile values, while the circle represents the mean value, and the bar across the box represents the 50^th^-percentile value. The whiskers outside the box represent the 10^th^- to 90^th^-percentile values.

**Figure 3:**
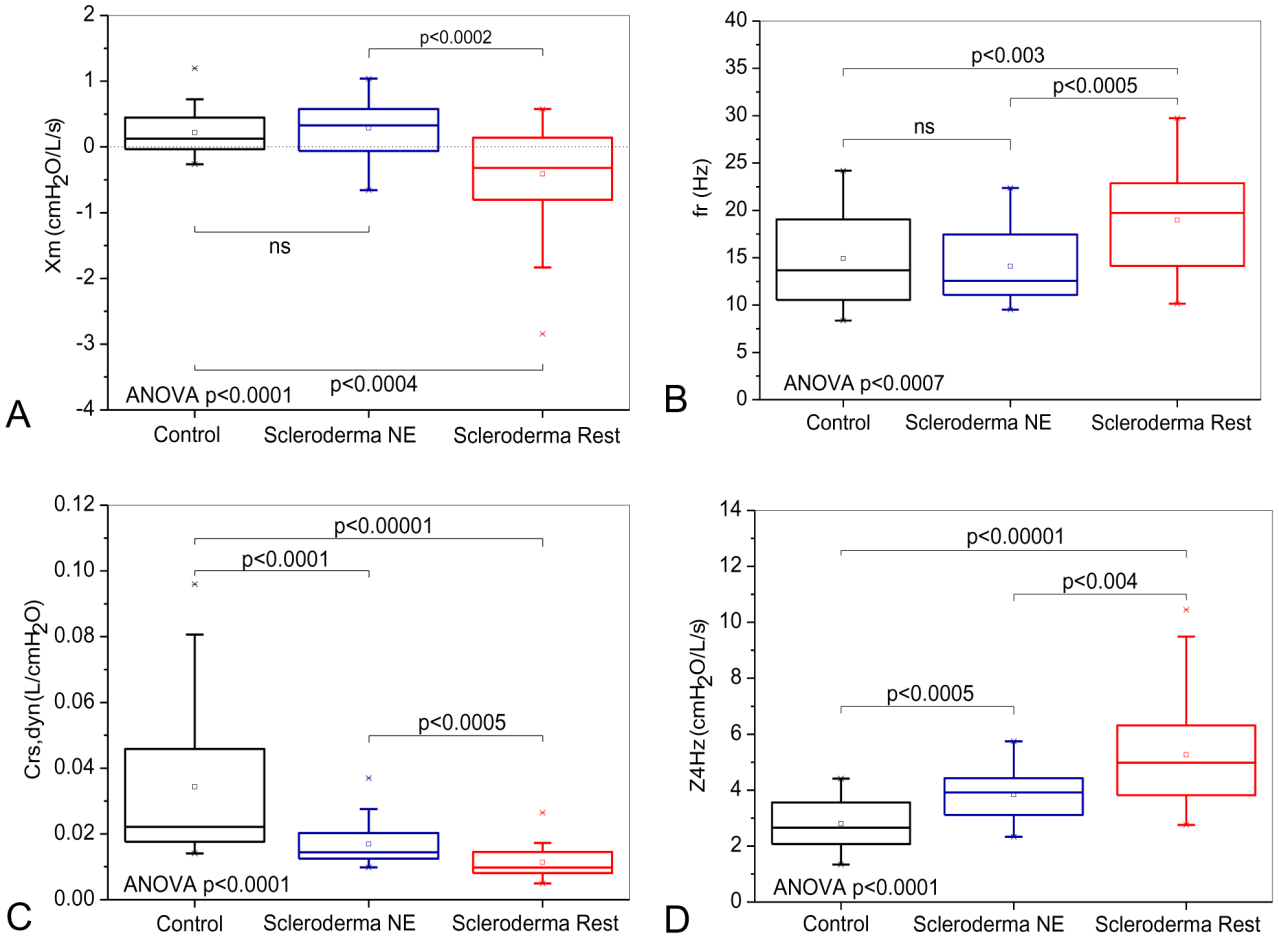
Influence of the pattern classified as restrictive in the spirometric exams on the reactive properties of the respiratory system. Mean reactance (Xm; Figure A) and the dynamic compliance of the respiratory system (Crs,dyn; Figure B) decreased, whereas the resonant frequency (fr; Figure C) and the impedance modulus at 4 Hz (Z4Hz; Figure D) exhibited significant increases. Notably, there are significant alterations in Crs,dyn and Z4Hz even in the patients classified as normal (Figures C and D).

Figures 2 and 3 depict the results of the comparative analysis of the resistive and reactive parameters among the groups classified according to spirometry. Significant increases of Ri, R4Hz and Rm were observed (Figures 2A, B and C; ANOVA p<0.001). A comparison among groups showed a significant difference between the control and normal test groups (p<0.01). A significant reduction of S was observed (Figure 2D; ANOVA p<0.004). A comparison among groups showed a significant difference between the normal exam and restrictive groups.

As presented in Figure 3A, SSc was found to have a significant impact on the Xm of the subjects, which was found to decrease (p<0.0001). Analysis of the subjects did reveal a statistically significant increase in fr (Figure 3B; p<0.0007) and also a decrease in Crs,dyn (Figure 3C; p<0.0001). The analysis of Crs,dyn showed a significant difference in the comparison among all the groups. The introduction of restriction in patients with SSc resulted in a significant increase of Z4Hz (Figure 3D; p<0.0001) with a significant difference in the comparison among all groups.

**Figure 4:**
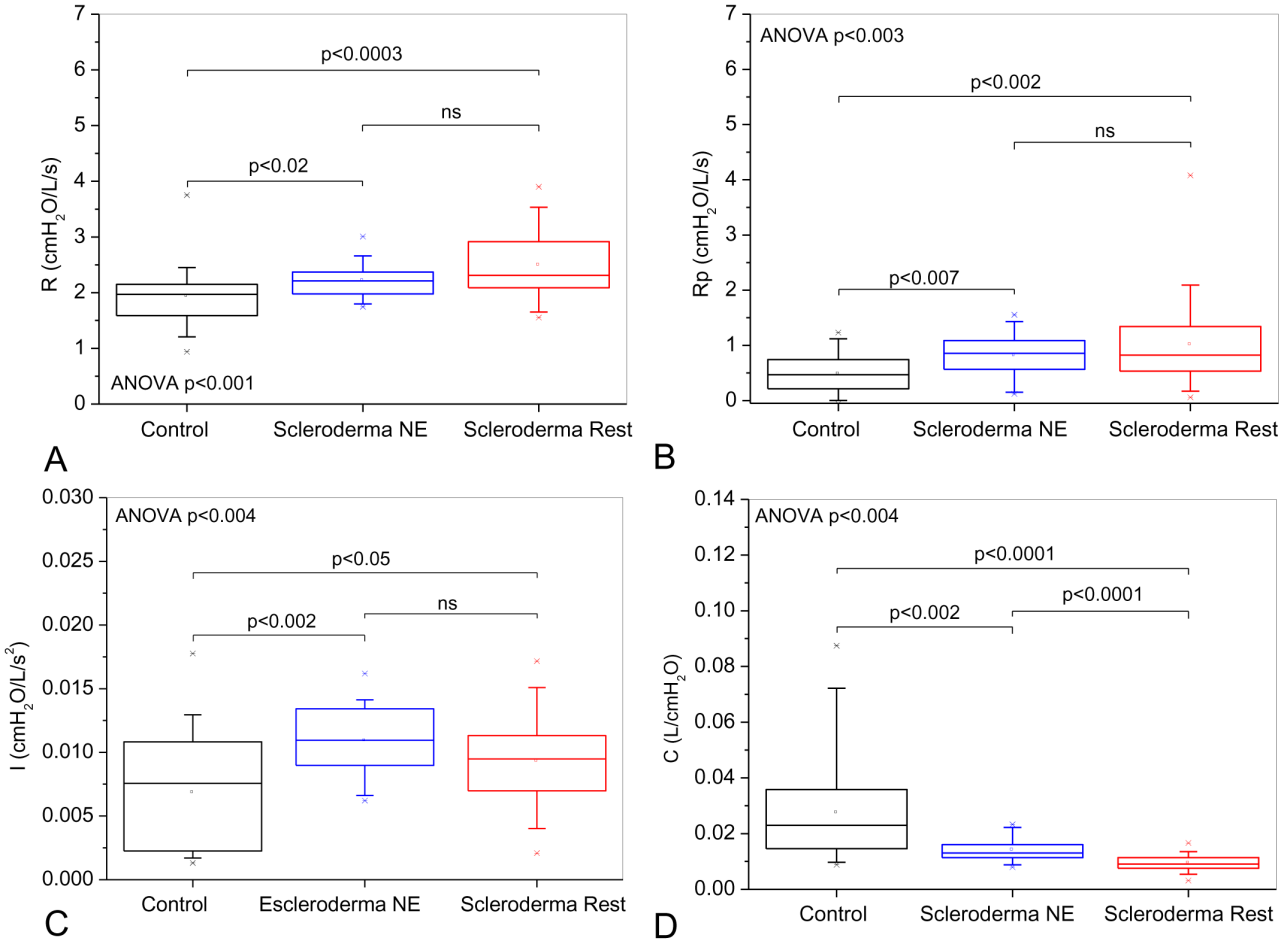
Influence of the pattern classified as restrictive in the spirometric exams on parameter values estimated from the model described in Figure 1. The increasing restriction was accompanied by a correspondent gradual elevation of central (A) and peripheral (B) resistance values. These changes are significant even in patients with normal spirometry. Respiratory inertance (C) increased in SSc. The changes observed in resistance and inertance may be explained by reductions in the radius of the airways. As can be observed in (D), SSc also resulted in reduced values of compliance.

Figure 4 show the influence of the pattern classified as restrictive in the spirometric exams on the eRIC model's parameter. A patient from the restrictive group that presented an outlier value of Rp = 27174 was removed of this analysis. The biometric homogeneity was not modified by removing this patient. Mean total errors in the model estimates for the entire data set was 0.12±0.10 cmH_2_O/L/s. Significant increases of R, and Rp were observed (Figures 4A and B; ANOVA p<0.003). A comparison among groups showed a significant difference between the control and normal test groups (p<0.02). A significant increase in I was observed (Figure 4C; ANOVA p<0.004). A comparison among groups showed a significant difference between the normal exam and restrictive groups (p<0.002). C decreased significantly with restriction (Figure 4D: ANOVA p<0.004).

**Figure 5:**
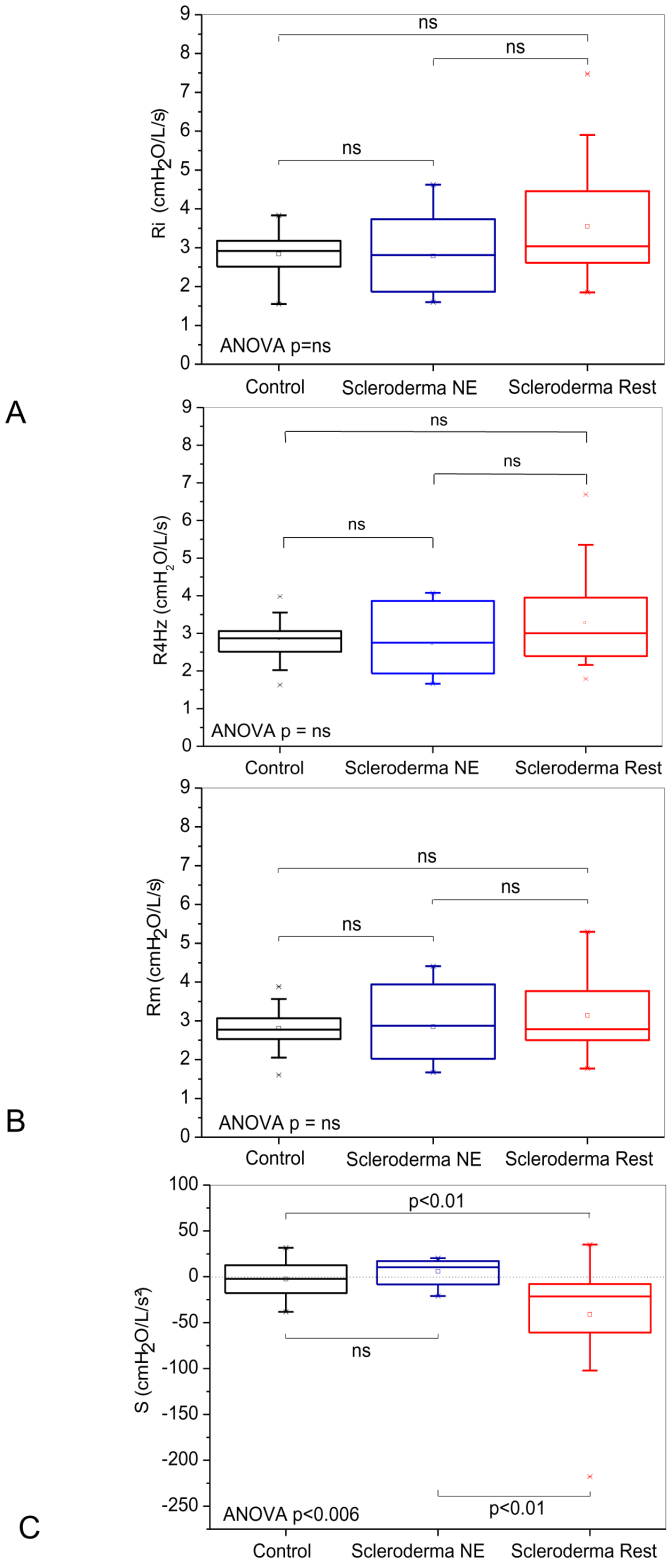
Effect of the restricted pattern in the lung volume tests on the resistive properties of the respiratory system. The total resistance of the respiratory system (Ri; Figure A) and the mean resistance (Rm; Figure B) did not change, whereas in (C), we observe that the restriction results in more negative S values.

**Figure 6:**
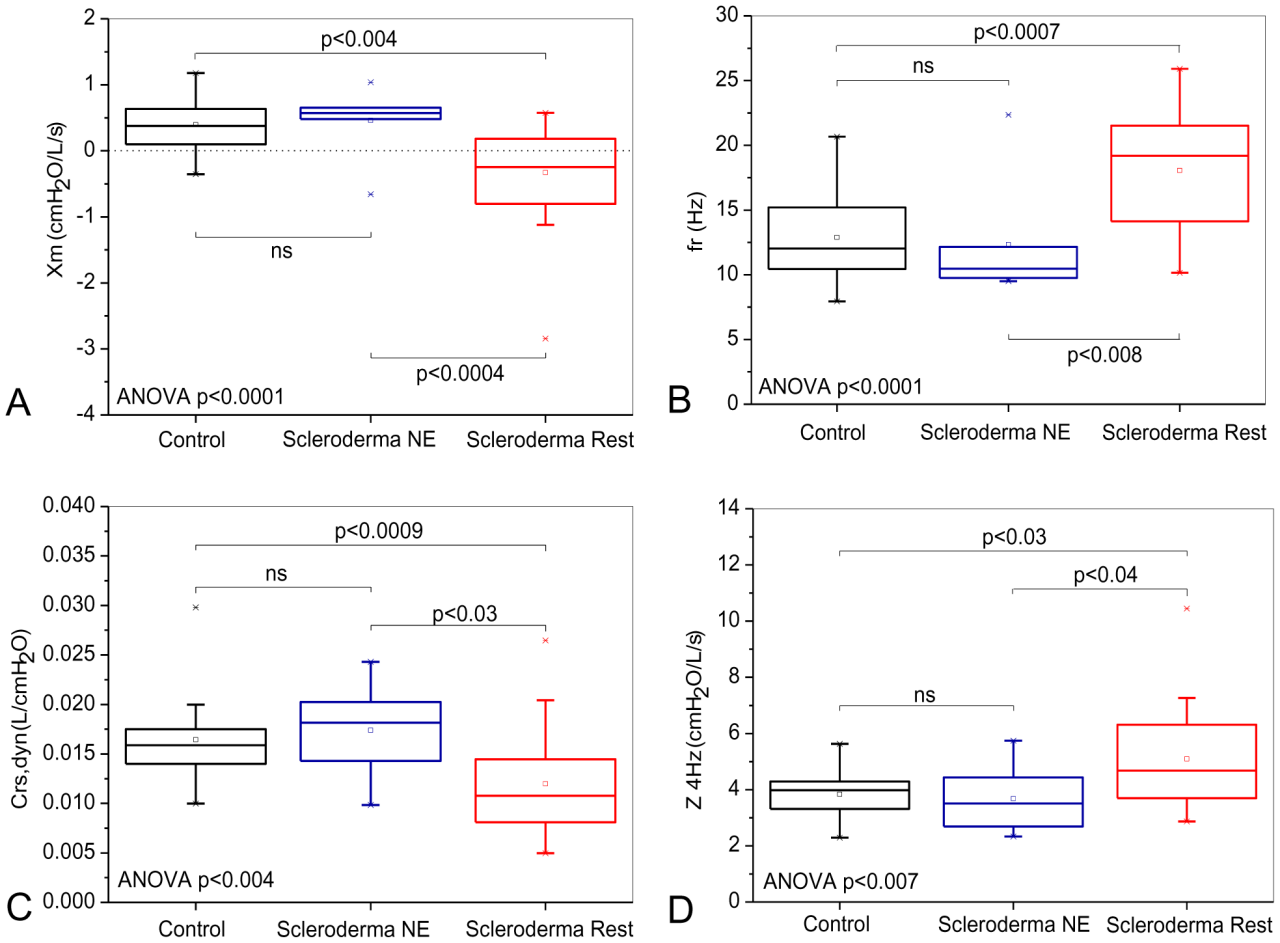
Influence of the restricted pattern in the lung volume tests on the reactive properties of the respiratory system. The mean reactance (Xm; Figure A) and the dynamic compliance of the respiratory system (Crs,dyn; Figure B) decreased, whereas the resonant frequency (fr; Figure C) and the impedance modulus at 4 Hz (Z4Hz; Figure D) exhibited a significant increase.

Figures 5 and 6 depict the results of the comparative analyses of the resistive and reactive parameters in the groups classified according to the lung volume analysis. No significant alterations were observed in Ri, R4Hz and Rm (Figures 5A, B and C). More negative values of S were observed with the restriction (Figure 5D; ANOVA p<0.006).

Taking into account the reactive parameters, we observed that the presence of restriction resulted in more negative Xm values (Figure 6A; ANOVA p<0.0001), increased fr (Figure 6B; ANOVA p<0.0001), reduced Crs,dyn (Figure 6C; ANOVA, p<0.004) and increased Z4Hz (Figure 6D; ANOVA p<0.007).

**Figure 7:**
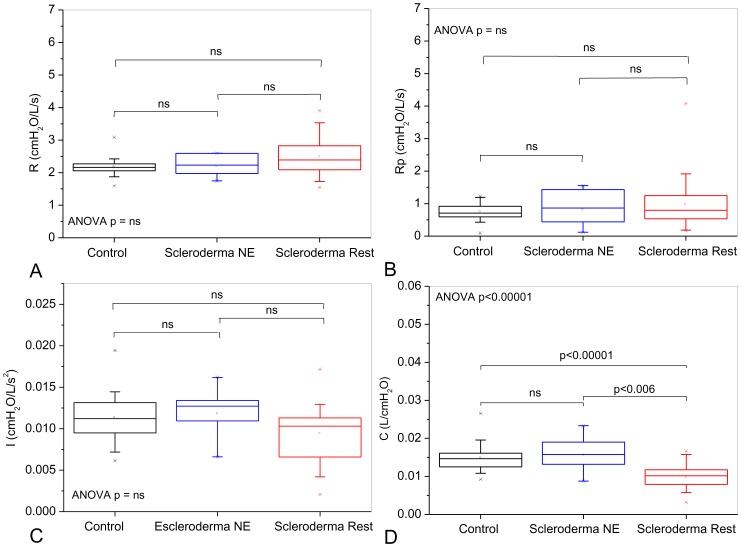
Influence of the restricted pattern in the lung volume tests on parameter values estimated from the model described in Figure 1. Changes in central (A) and peripheral resistance (B), as well as in I (C) values were non-significant. Compliance was significantly reduced in restrictive patients (D).

The changes on the eRIC model's parameter associated with the pattern of restriction in the lung volume tests are described in Figure 7. The mean total error in the model estimates was 0.17±0.19 cmH_2_O/L/s. There were no significant changes in R, Rp and I (Figures 7A, B and C; ANOVA p = ns). A significant reduction was observed in C (Figure 4D; ANOVA p<0.001).

### Correlation among the forced oscillations, spirometry and lung volume parameters

Ri exhibited a reasonable and inverse correlation with all the parameters used in the spirometric classification, except for the variable FEF/FVC (Table 4). A similar behavior was exhibited by Rm. S exhibited a reasonable direct correlation with all the spirometric parameters, except for the FEV_1_/FVC and FEF/FVC ratios. Xm exhibited a good direct correlation with FEV_1_ and FVC. Fr exhibited a reasonable inverse correlation with FEV_1_, FVC and FEF_25–75%_ (L), whereas Crs,dyn exhibited a reasonable direct correlation with FEV_1_, FVC and FEV_1_/FVC. Z4Hz exhibited a moderate to good inverse correlation with FEV_1_ (%) and FVC (%).

**Table 4:** Correlation coefficient (R) and significance level (p) of the analysis including FOT and spirometric exams.

		Ri (cmH_2_O/L/s)	R4Hz (cmH_2_O/L/s)	S (cmH_2_O/L/s^2^)	Rm (cmH_2_O/L/s)	Xm (cmH_2_O/L/s)	fr (Hz)	Crs,dyn (L/cmH_2_O)	Z4Hz (cmH_2_O/L/s)	R (cmH_2_O/L/s)	Rp (cmH_2_O/L/s)	I (cmH_2_O/L/s^2^)	C (L/cmH_2_O)
FEV_1_ (L)	R	−0.38	−0.34	0.46	−0.28	0.52	−0.47	0.28	−0.48	−0.20	−0.36	0.05	0.30
	p	0.004	0.001	0.0001	0.01	0.0001	0.001	0.01	0.0001	0.078	0.0009	0.65	0.006
FEV_1_ (%)	R	−0.42	−0.39	0.42	−0.35	0.45	−0.38	0.41	−0.52	−0.31	−0.39	−0.08	0.32
	p	0.0001	0.0003	0.001	0.001	0.0001	0.004	0.0001	0.0001	0.006	0.0003	0.49	0.003
FVC(L)	R	−0.38	−0.35	0.45	−0.28	0.51	−0.46	0.29	−0.49	−0.22	−0.36	0.03	0.33
	p	0.004	0.001	0.0001	0.01	0.0001	0.001	0.008	0.0001	0.04	0.0008	0.79	0.003
FVC (%)	R	−0.40	−0.39	0.42	−0.33	0.47	−0.39	0.38	−0.52	−0.30	−0.37	−0.05	0.32
	p	0.001	0.0003	0.0001	0.002	0.0001	0.002	0.0004	0.0001	0.006	0.0007	0.63	0.003
FEV_1_/FVC	R	−0.29	−0.26	0.08	−0.34	−0.39	0.07	0.35	−0.28	−0.25	−0.22	−0.32	0.28
	p	0.006	0.02	0.46	0.001	0.0002	0.51	0.001	0.009	0.02	0.05	0.003	0.01
FEF_25–75%_(L)	R	−0.28	−0.25	0.34	−0.20	0.36	−0.29	0.08	−0.29	−0.10	−0.25	0.12	0.04
	p	0.01	0.03	0.001	0.06	0.0009	0.007	0.45	0.007	0.37	0.03	0.29	0.70
FEF_25–75%_(%)	R	−0.30	−0.28	0.26	−0.27	0.23	−0.14	0.20	−0.31	−0.18	−0.29	−0.04	0.06
	p	0.005	0.01	0.02	0.01	0.03	0.22	0.06	0.005	0.10	0.009	0.73	0.62
FEF/FVC	R	0.08	0.08	−0.14	0.04	−0.16	0.18	−0.19	0.19	0.10	0.18	0.09	−0.27
	p	0.49	0.45	0.22	0.73	0.16	0.09	0.07	0.09	0.36	0.10	0.43	0.01

Considering the correlations between the FOT parameters and those used in the classification based on volumes (Table 5), we observe that Ri exhibited a significant correlation only with TLC (L). Rm did not exhibit a significant correlation with the lung volumes parameters, and S only correlated weakly with TLC (L) and RV/TLC. The greatest correlations were observed between parameters Xm, fr and Crs,dyn and TLC (L). Zrs exhibited an inverse correlation with TLC and RV (L).

**Table 5:** Correlation coefficient (R) and significance level (p) of the analysis including FOT and pulmonary volumes exams.

		Ri (cmH_2_O/L/s)	R4Hz (cmH_2_O/L/s)	S (cmH_2_O/L/s^2^)	Rm (cmH_2_O/L/s)	Xm (cmH_2_O/L/s)	fr (Hz)	Crs,dyn (L/cmH_2_O)	Z4Hz (cmH_2_O/L/s)	R (cmH_2_O/L/s)	Rp (cmH_2_O/L/s)	I (cmH_2_O/L/s^2^)	C (L/cmH_2_O)
TLC (L)	R	−0.34	−0.28	0.39	−0.24	0.52	−0.53	0.55	−0.48	−0.25	−0.36	0.26	0.69
	p	0.02	0.05	0.004	0.09	0.0001	0.001	0.0001	0.0004	0.08	0.03	0.07	0.0001
TLC (%)	R	−0.22	−0.17	0.26	−0.16	0.41	−0.48	0.43	−0.35	−0.22	−0.17	0.23	0.53
	p	0.12	0.23	0.07	0.28	0.003	0.003	0.002	0.01	0.11	0.23	0.11	0.0001
RV (L)	R	−0.20	−0.22	0.11	−0.21	0.26	−0.29	0.36	−0.29	−0.18	−0.21	0.09	0.40
	p	0.16	0.12	0.43	0.14	0.07	0.03	0.01	0.04	0.20	0.14	0.52	0.004
RV (%)	R	−0.13	−0.14	0.07	−0.14	0.21	−0.27	0.28	−0.22	−0.18	−0.10	0.11	0.29
	p	0.36	0.32	0.60	0.33	0.14	0.06	0.04	0.13	0.21	0.46	0.45	0.04
RV/TLC	R	0.15	0.06	−0.34	0.01	−0.26	0.16	−0.12	0.15	0.06	0.12	−0.20	−0.21
	p	0.29	0.67	0.02	0.93	0.07	0.27	0.42	0.29	0.66	0.40	0.19	0.15

### Assessment of the clinical potential of forced oscillation parameters

Table 6 describes the values of the area under the curve (AUC), sensitivity (Se), specificity (Sp) and the cut-off points of the comparisons between the control and restrictive groups according to the classifications based on spirometry and lung volumes.

**Table 6:** Values of area under the curve (AUC), sensitivity (Se) and specificity (Sp) for the optimal cut-off points for the FOT indices describing the performance of FOT parameters in the detection of patients considered restrictive according to the spirometric and volumetric classifications.

		AUC	Se (%)	Sp (%)	Cut-off
Ri	Spirometry	0.81	73.3	66.7	2.61
(cmH_2_O/L/s)	Volumes	0.63	54.5	47.6	2.91
R4Hz	Spirometry	0.80	80.0	60.0	2.39
(cmH_2_O/L/s)	Volumes	0.57	59.1	52.3	2.87
Rm	Spirometry	0.79	70.0	66.7	2.50
(cmH_2_O/L/s)	Volumes	0.57	54.5	52.4	2.78
S	Spirometry	0.72	63.3	66.7	−12.17
(cmH_2_O/L/s^2^)	Volumes	0.73	63.6	61.9	−10.91
Xm	Spirometry	0.77	63.3	66.7	−0.01
(cmH_2_O/L/s)	Volumes	0.82	68.2	66.7	0.11
fr	Spirometry	0.73	63.3	66.7	17.20
(Hz)	Volumes	0.81	68.2	61.9	14.13
Crs.dyn (L/cmH_2_O)	Spirometry	0.95	86.7	90.0	0.015
	Volumes	0.80	72.7	66.7	0.014
Z4Hz (cmH_2_O/L/s)	Spirometry	0.91	83.3	80.0	3.58
	Volumes	0.71	63.6	61.9	3.99
R	Spirometry	0.78	75.9	63.3	2.09
(cmH_2_O/L/s)	Volumes	0.65	59.1	66.7	2.22
Rp	Spirometry	0.74	72.4	60.0	0.54
(cmH_2_O/L/s)	Volumes	0.53	54.5	52.4	0.71
I	Spirometry	0.65	62.1	56.7	0.008
(cmH_2_O/L/s^2^)	Volumes	0.66	54.5	66.6	0.010
C	Spirometry	0.94	89.7	86.7	0.013
(L/cmH_2_O)	Volumes	0.86	63.6	90.5	0.011

AUCs considered adequate for clinical use (≥ 0.80) are described in yellow.

### Expiratory flow limitation analysis

**Figure 8:**
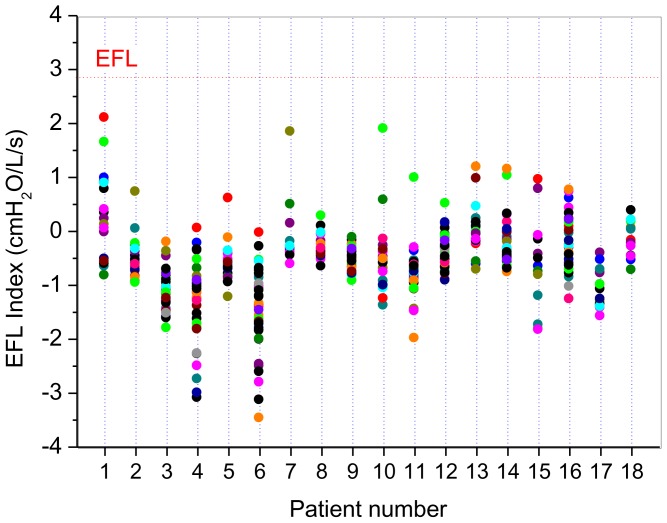
Expiratory flow limitation (EFL) index in patients with systemic sclerosis. EFL events are described by values above the threshold value plotted as a horizontal line. None of the 411 breaths cycles analyzed in the eighteen studied subjects had expiratory flow limitation as defined by an EFLi > 2.8 cmH_2_O/L/s. These results provide evidence that EFL during spontaneous breathing is not common in patients with SSc.

The anthropometric characteristics of the investigated patients are described in Table 7. Eighteen patients were studied, 15 of them were never-smokers and 3 were light smokers (5,6, and 7 pack-years). It was analyzed a total of 411 breaths cycles. Figure 8 show all values of the EFLi for each studied patient. None of the eighteen studied subjects presented an EFLi *>* 2.8 cmH_2_O/L/s.

**Table 7:** Anthropometric characteristics of the patients studied in the analysis of expiratory flow limitation.

		Mean (SD)
Male/Female		2/16
Age (years)		50.1±13.9
Body mass (kg)		66.0±11.2
Height (cm)		160.5±5.7
FVC (L)		2.2±0.6
FVC (%)		70.0±13.3
FEV_1_ (L)		1.7±0.5
FEV_1_ (%)		71.1±15.3
FEV_1_/FVC		83.0±5.8
FEF_25–75%_ (L)		2.2±1.0
FEF_25–75%_ (%)		83.9±40.2
FEF/FVC		106.8±43.2

## Discussion

Initially, in a multi-frequency analysis, the present study investigated the influence of the restrictive pattern that develops in SSc on the resistive and restrictive properties of the respiratory system. These analyses were performed using the two most typical methods of respiratory analysis in these patients, namely, spirometry and lung volume assessment. Thus, it was shown that in these patients, Rrs is increased, and Xrs becomes more negative, with the exhibited alterations being proportional to the intensity of restriction. A compartmental model analysis showed increased values of central and peripheral resistances. Importantly, the alterations of FOT parameters were correlated with the standard methods of analysis of the pulmonary function. ROC analysis showed that seven FOT parameters exhibited a great potential for assisting the diagnosis of the respiratory effects of scleroderma. In addition, a mono-frequency study demonstrated that EFL during spontaneous breathing is not common in patients with SSc.

### Analyzed groups

The analyzed groups can be considered quite homogeneous (Table 1). In the classification based on lung volumes, the only biometric parameter that was different upon comparison among the investigated groups was age in the classification based on lung volumes. This fact, however, is no cause of concern because ageing do not significantly alter the FOT parameters, whereas height is the most influential parameter [20], [58].

As expected, the changes in the spirometric parameters (Table 2) agree with the classification of restrictive disorder by means of spirometry [34], [35]. An FEV_1_/FVC ratio above 70% denotes a lack of airflow obstruction [53]. During the study stage that focused on the classification according to the lung volumes, the restrictive group exhibited a reduction of all the volumetric parameters compared to the remaining groups, except for the RV/TLC ratio (Table 3). These findings support the classification of restrictive disorder by means of the helium dilution technique [34], [35].

The use of FOT allowed us a detailed analysis of the respiratory system in systemic sclerosis. The changes in the resistive and reactive properties observed in these patients and the possible mechanisms behind these pathophysiological changes are discussed in the following sections.

### Resistive parameters

We found an increase of Ri and R4Hz (Figure 2A, and B, respectively). These results might be explained by a development of peribronchial fibrosis that is affecting the bronchial wall, which contributes to a reduction of the airway caliber [8], [59]–[62]. In agreement with our results, Blom-Bülow *et al.* [52] reported an increase of the pulmonary resistance by 33% in patients with SSc, and they attributed this finding to an increase of the viscous resistance of the lung tissue combined with peribronchial fibrosis. Autopsy studies performed in patients with SSc found changes characteristic of increased resistance, such as atrophy and fibrosis of the muscular and elastic fibers of the bronchia, in addition to peribronchial fibrosis [63].

We found an increase of Rm when we considered the classification based on spirometry (Figure 2C). However, we did not find modifications of this parameter with the classification based on the lung volumes (Figure 5C). Possibly, the results described in Figure 2C are due to extensive peribronchial fibrosis, which causes a reduction of the internal airway diameter in patients with SSc [8], [59], [61]–[64]. A further relevant factor might be the fibrotic process that results in a reduction of the lung volumes and a consequential anatomical narrowing of the airway [64]. According to Oor *et al.* [65], SSc affects the small and the large airways in 45–100% of patients. In addition, Guttadauria *et al.* [64] pointed to the occurrence of structural alterations in SSc, such as atrophy of the bronchial wall smooth muscle, and a loss of the supporting elastic tissue, which might contribute to the increased airway resistance.

In contrast to Ri, R4Hz and Rm, which changed their behavior as a function of the different classifications used (Figures 2A, 2B, 2C and Figures 5A, 5B and 5C), S became more negative in the presence of restriction that was independent of the classification used (Figures 2C and 5C). This parameter is associated with the homogeneity of the respiratory system [42], [43]. The reduced homogeneity of the restrictive group (Figures 2C, 5C) is consistent with the physiological alterations caused by SSc because the functional changes in the lungs of these patients are probably related to the presence of variable degrees of interstitial fibrosis that cause volumetric restriction and of peribronchial fibrosis that causes air trapping [60], [64]. In addition, the intensive replacement of lung parenchyma by fibrous tissue and the thickening of the alveolar septum might cause local alterations of the elasticity and, consequently, an imbalance in the time constants of the respiratory system, which contribute to the increase of non-homogeneity [66].

A compartmental model analysis can contribute to a better knowledge of the physiopathology of the SSc. Considering the influence of the pattern classified as restrictive in the spirometric exams on the eRIC model's parameter, it was initially observed that this analysis resulted in resistance values that are in line with what is expected in patients with mild airflow obstruction (Figure 4A and B). The increasing restriction was accompanied by a correspondent gradual elevation of resistances. These results were consistent with that described in Table 2 and Figure 2. Figure 4 also show that SSc introduces an increase in both, central and peripheral resistances, and that these changes are relevant even in patients with normal spirometry. The compartmental model analysis associated with the pattern of restriction in the lung volume tests resulted in non-significant changes in central and peripheral resistance values (Figure 7A,B).

An analysis of the correlation between the resistive and spirometric parameters (Table 4) showed that Ri, R4Hz, Rm and S exhibited similar behaviors with predominantly reasonable correlations. The highest values of the coefficient of correlation were exhibited by FEV_1_, which allowed us to infer that these parameters represent mainly alterations of the central airways. When studying patients with sarcoidosis, Faria *et al.* [25] found similar results. Table 4 also shows that R was associated with spirometric parameters representing mainly alterations of the central airways (FEV_1_, FEV_1_/FVC) and pulmonary volume (FVC), while Rp is also related to small airways (FEF_25–75%_).

### Reactive parameters

Whereas S is associated with non-homogeneity in terms of resistance distribution, Xm describes non-homogeneity in terms of the reactive properties of the respiratory system. The restrictive group exhibited more negative values of Xm, thereby denoting greater non-homogeneity compared to the control and normal to the exam groups (Figures 3A and 6A). These results were similar in both types of classification investigated and are possibly due to the progressive process of diffuse pulmonary interstitial fibrosis that is inherent to the physiopathology of SSc [6], [7], [15], [60], [62]. That process appears to be the cause for the establishment of hypoventilated areas and the promotion of atelectasis in addition to important alterations of the time constants [60].

The resonancefrequency is associated with the inertial and elastic properties of the respiratory system and is an important sign of changes in homogeneity. Independent of the classification method used, the restrictive group exhibited higher values of fr. Thus indicating greater non-homogeneity and the presence of a more fibrotic tissue compared to the control and normal to the exam groups (Figure 3B and 6B).

Figures 3C and 6C show a significant reduction of Crs,dyn in the presence of restriction. As can be observed in Figures 4D and 7D, the analysis based on the eRIC model also resulted in reduced values of compliance in patients with SSc. These results clearly reflects the physiopathology of SSc, which includes diffuse fibrosis affecting the interstitium and alveolar septa, thereby changing their architecture and the bronchial wall [6]–[8], [12], [15], [19], [59], [62], [63], [66]. This accurate description of the physiopathology of SSc afforded by Crs,dyn allows us to infer that the FOT might be an alternative method to quantitatively and non-invasively assess the elastic properties of the respiratory system in these individuals, thus avoiding the use of invasive techniques, such as that based on esophageal balloon.

Van Noord *et al.* [67] suggested that tissue resistance is inversely proportional to lung compliance. Our results agree with that suggestion because resistances increased (Figure 2A,B,C) when Crs,dyn decreased (Figures 3C, 4D). Upon studying kyphoscoliosis and ankylosing spondylitis, which are disorders of the chest wall, Van Noord *et al.* [68] concluded that the compliance of the chest wall was reduced, but that its resistance was increased. The results depicted in Figures 2A and 3C agree with those authors.

Boros *et al.* [69] attributed the increase of elastance with a displacement of the elastic equilibrium, which points to a reduction of the lung volumes associated with interstitial diseases. Our results agree with this assertion because Crs,dyn, which varies inversely and proportionally to elastance, was considerably reduced (Figure 3C and 6C). Those authors also suggested that a reduction of the lung static compliance was one of the early alterations in these diseases. In our study, the normal to the exam group exhibited a significant reduction of the Crs,dyn compared to the control group (Figure 3C), thus supporting the hypothesis proposed by those authors, who also used spirometry as a reference.

Using the esophageal balloon technique, Blom-Bülow *et al.* [61] and Greenwald *et al.* [12] found a reduction of the lung static compliance in all the patients with SSc. When we studied the dynamic compliance of the respiratory system, we observed a similar behavior (Figures 3C, 6C, 4D and 7D). In spite of the different measurement methods, the static and dynamic compliances did not exhibit perceptible differences in the healthy individuals [70]. In individuals with respiratory diseases, the relationship between the static and dynamic compliances was altered by an increase of the respiratory rate. In these individuals, the dynamic compliance was reduced due to alterations in the airway resistance and the alveolar compliance, thereby making alveolar inflation difficult and contributing to the increase of the time constants. Together, these alterations result in poorly functioning alveolar units, which further reduce the dynamic compliance [70].

The estimates of respiratory inertance using the eRIC model showed increased values of inertance in SSc patients classified using spirometry (Figure 4C). Respiratory inertance reflects mainly the mass of central airway gas. Since resistance and inertance are inversely proportional to the radius of a tube [51], it might be expected that the increase in airway obstruction observed in Figures 2 and 4 would be accompanied by a higher inertance. Figure 7C shows that the respiratory inertance do not changed in SSc patients classified according to the lung volumes. This behavior may be explained by the absence of airway obstruction (Figures 5 and 7) in this classification.

Z4Hz increased with the presence of restriction (Figure 2D and 4D). This parameter is related with the total mechanical load of the respiratory system, including the resistive and elastic effects observed in 4 Hz. These results are consistent with the physiopathology of SSc because we found alterations in both the resistive and reactive properties of the respiratory system [6]–[8], [15], [19], [59], [60], [62], [63], [66]. The increase of Z4Hz reflects an increase in respiratory effort, which might be the origin of dyspnea and the consequential reduction of the quality of life that is usually reported by patients diagnosed with SSc.

In the analysis of the correlation between the reactive and spirometric parameters (Table 4), fr and Crs,dyn exhibited similar behaviors with a predominantly reasonable correlation. The greatest correlations were exhibited by FEV_1_, which suggests that these parameters best represent alterations in the central airways. Xm exhibited good correlation with FEV_1_ (L) and FVC (L), thereby expressing more extensively the alterations in the central airways and the reduction of the lung volumes. Z4Hz exhibited a good correlation with FEV_1_ (%) and FVC (%). Therefore, Z4Hz seems to reflect alterations of the central airways and the reduction of the lung volumes that are inherent to the physiopathology of SSc. Considering the reactive parameters obtained by the model analysis, Table 4 shows that I was associated with FEV_1_/FVC, while C presented reasonable correlations with FEV_1_ and FVC.

In general, the FOT parameters exhibited reasonable correlations with the spirometric parameters, which disagrees with the findings by Van Noord *et al.* [67] on patients with diffuse interstitial lung disease, where the analyzed spirometric parameters (FVC and FEV_1_) exhibited excellent correlations with the FOT parameters. However, a later study by those same authors, Van Noord *et al.* [68], that analyzed patients with kyphoscoliosis and ankylosing spondylitis also found low correlation values. Faria *et al.* [25] also found low correlations between the FOT and spirometric parameters in patients with sarcoidosis, and they at least partially attributed this result to the methodological differences between the tests. These differences are associated the use of quiet respiration by the FOT while spirometry employs forced maneuvers.

It was observed that the best associations among FOT and pulmonary volumes exams were obtained among Xm, fr and Crs,dyn and TLC (Table 6). This Table also shows that the best association was between C obtained by the model analysis and TLC. These results indicated good associations between these parameters, providing additional support to the hypothesis that FOT may provide a non-invasive alternative to the evaluation of the elastic properties of the respiratory system in SSc patients.

### Evaluation of the diagnostic use of the forced oscillation parameters

The parameters assessed in the present study were able to describe consistently the physiopathology of systemic sclerosis, and several values were different from those of the healthy individuals (Figures 2 to 7). This is an important fact because it shows that these parameters are potentially useful for the diagnosis of lung function alterations caused by SSc. Taking into account that the FOT exam is easily performed, we might suppose that the FOT might represent a significant contribution to the simplification of the diagnosis of the lung function affection in SSc. The analysis of the ROC curve showed that 7 of the 12 studied parameters exhibited adequate diagnostic accuracy (Table 6).

The ROC curves plots the probability of true-negative (specificity) versus the probability of false-positive (1-sensitivity) for various decision criteria. This way, the larger the AUC, more valid the diagnostic test in comparison with the gold standard. An area under the ROC curve ≥ 0.80 is widely accepted as adequate for diagnostic use [57], [71]. In this analysis, Ri, R4Hz exhibited a value useful for clinical diagnostic in regard to the spirometric classification (AUC>0.80), whereas the values of S and Rm were unsatisfactory (AUC<0.80). Xm and fr exhibited values useful for clinical application according to the classification based on lung volumes (AUC = 0.82 and 0.81, respectively).

Compliance obtained using the eRIC model, Crs,dyn and Z4Hz stood out because of their high values of AUC (>0.90), which represent a high level of accuracy [57], [71]. It was interesting to observe that the changes in resistive parameters were relatively low (Figure 1) comparing with that observed in obstructive diseases as COPD [23] or asthma [24]. The observed findings are in close agreement with the pathophysiology of SSc. In contrast with COPD and asthma, this disease is associated with a restrictive pattern. This may explain why reactive parameters presented a better diagnostic performance than the resistive ones.

Gupta *et al.* [16] assessed 10 patients using an esophageal balloon and plethysmography and found that static compliance and transpulmonary pressure are good parameters for the early identification of lung affection in SSc. Despite the difference in the methods, we may observe that the results of the present study are consistent with the abovementioned study because they show that the parameters related to the elastic properties of the respiratory system exhibit a better diagnostic performance compared to the parameters corresponding to the resistive properties.

### Expiratory Flow Limitation Analysis

Expiratory flow limitation during tidal breathing in patients with severe obstructive pulmonary disease is a well-recognized phenomenon [32], [33]. It may be present, however, also in patients with severe chest wall disease due to the fact that breathing takes place at reduced lung volume [32]. Figure 8 shows that none of the 411 breaths cycles analyzed in the eighteen studied subjects had expiratory flow limitation as defined by an EFLi *>* 2.8 cmH_2_O/L/s. Such analysis provides useful information in SSc, and shows that none of the patients with SSc were flow limited. These results are coherent with previous studies [32], and confirms that EFL during spontaneous breathing is not common in patients with restrictive respiratory disorders.

### Limitations of the study

Similar to other techniques of functional assessment, the FOT limitations and consequences must be recognized. During the exams, part of the oscillatory flow is shunted by the impedance of the compliant cheeks and pharynx [20]–[22], [44], which are placed in parallel with the respiratory system. The resulting effect is to reduce the impedance measured in relation to its actual value. This effect becomes progressively stronger as the respiratory resistance increases, as is the case for highly obstructive patients. Because we are studying patients with a relatively low resistance (Figures 2 and 5) compared with typical obstructive patients [23], [24], it does not represent a notable problem in the present study. In addition, the shunt effect was minimized by asking the patient to firmly support their cheeks and mouth floor [20]–[22], [51].

The process of spontaneous breathing introduces both random and systematic errors [20]–[22], [51]. In the present study, these errors are reduced with excitation frequencies (4–32 Hz) at least 20 times higher than those present in the spontaneous ventilation process (≅0.2 Hz). These errors may be easily evaluated using the coherence function (γ^2^) between the pressure and airflow signals [20], [23]–[26], [40]. A minimal coherence value of 0.9 is usually considered adequate [23]–[26], [40]. Any time the coherence computed for any of the studied frequencies is smaller than this threshold, the maneuver may not be considered valid, and the examination needs to be repeated.

The interpretation of FOT data in physiological terms demands the use of parameters describing lung structure and function, which are linked in a quantitative and anatomically representative way. Fitting mathematical models may help to obtain such parameters. However, there are numerous models, and different models may be fitted by the same impedance data [21], [51], [54]. The choice of the model depends largely on the experience of the authors. This fact introduces great difficulties in comparing results from different studies. In addition, more complicated models would not allow statistically reliable parameters estimates [72], [73]. Nonetheless, modeling is useful in distinguishing which mechanisms dominate the impedance data for a specific physiologic state or disease [22]. To minimize the problem of model complexity, a simple four-element compartmental model was used for interpreting forced oscillation measurements [54].

For a diagnostic tool it is important not only to detect changes between patients and controls, but also to be able to perform differential diagnosis. As pointed out before, the resistance changes in SSc were relatively low comparing with that observed in obstructive diseases [23], [24]. In addition, the Crs,dyn presented higher changes in SSc than in asthma [24]. We believe that these characteristics may help in the differential diagnosis and that the evaluation of this hypothesis deserves further studies.

### Synthesis with previous knowledge

Clinical pulmonary function tests are of prime importance for the diagnosis and monitoring of disease progression, and they feature centrally in the clinical practice for SSc [2], [3], [5], [9], [10], [12], [13], [15], [16]. The FOT is currently state-of-the-art for the assessment of lung function [21]. Although the important role of the FOT in the evaluation of respiratory diseases has been extensively documented [20]–[22], [27]–[31], [40], [46]–[51], [67], [68], [72]–[75] since its development over five decades ago [75] and despite the obvious advantages of the FOT in terms of noninvasiveness and a lack of dependence on patient cooperation, the FOT has still not become a standard methodology in the clinic [21]. It was noted that further data are required to allow these measurements to be used in clinical practice [76]. The present results provide the first direct experimental evidence demonstrating an important role for the FOT in the study of the SSc pathophysiology and in the diagnosis of respiratory abnormalities in a routine clinical setting.

## Conclusions

In this study, we present evidence that the resistive and reactive properties of the respiratory system are changed in SSc. This analysis contributed to the elucidation of pathophysiological fundamentals involved in this disease. Furthermore, these parameters are able to adequately detect alterations in the respiratory mechanics described by spirometric and volumetric exams. FOT is easy to perform and provides a detailed analysis of the respiratory system. These practical considerations, as well as the results of the present study, indicate that FOT may be a promising tool to facilitate the diagnosis of respiratory abnormalities in patients with SSc.
